# Sub-MICs of Azithromycin Decrease Biofilm Formation of *Streptococcus suis* and Increase Capsular Polysaccharide Content of *S. suis*

**DOI:** 10.3389/fmicb.2016.01659

**Published:** 2016-10-20

**Authors:** Yan-Bei Yang, Jian-Qing Chen, Yu-Lin Zhao, Jing-Wen Bai, Wen-Ya Ding, Yong-Hui Zhou, Xue-Ying Chen, Di Liu, Yan-Hua Li

**Affiliations:** ^1^College of Veterinary Medicine, Northeast Agricultural UniversityHarbin, China; ^2^Heilongjiang Academy of Agricultural SciencesHarbin, China

**Keywords:** proteomics, *Streptococcus suis*, azithromycin, biofilm formation, capsular polysaccharide

## Abstract

*Streptococcus suis* (*S. suis*) caused serious disease symptoms in humans and pigs. *S. suis* is able to form thick biofilms and this increases the difficulty of treatment. After growth with 1/2 minimal inhibitory concentration (MIC) of azithromycin, 1/4 MIC of azithromycin, or 1/8 MIC of azithromycin, biofilm formation of *S. suis* dose-dependently decreased in the present study. Furthermore, scanning electron microscopy analysis revealed the obvious effect of azithromycin against biofilm formation of *S. suis*. Especially, at two different conditions (1/2 MIC of azithromycin non-treated cells and treated cells), we carried out comparative proteomic analyses of cells by using iTRAQ technology. Finally, the results revealed the existence of 19 proteins of varying amounts. Interestingly, several cell surface proteins (such as ATP-binding cassette superfamily ATP-binding cassette transporter (G7SD52), CpsR (K0FG35), Cps1/2H (G8DTL7), CPS16F (E9NQ13), putative uncharacterized protein (G7SER0), NADP-dependent glyceraldehyde-3-phosphate dehydrogenase (G5L259), putative uncharacterized protein (G7S2D6), amino acid permease (B0M0G6), and NsuB (G5L351)) were found to be implicated in biofilm formation. More importantly, we also found that azithromycin affected expression of the genes *cps1/2H, cpsR* and *cps16F*. Especially, after growth with 1/2 MIC of azithromycin and 1/4 MIC of azithromycin, the capsular polysaccharide content of *S. suis* was significantly higher.

## Introduction

*Streptococcus suis* (*S. suis*) causes serious disease symptoms. It is one of the most important pathogens, and it is responsible for pneumonia, arthritis, septicemia, and meningitis in pigs and humans (Bojarska et al., 2016). So far, some researchers have reported that *S. suis* is able to form biofilms (Wang et al., 2015; Bojarska et al., 2016; Espinosa et al., 2016). Biofilm structures are packaged along with proteins, nucleic acids, extracellular polysaccharides (EPS), and other materials. Microbial cells within biofilm structures are tolerant to the host immune system, heat treatment, and antibiotics (Nakamura et al., 2016).

In accordance with the antigenicity of the capsule, the *S. suis* serotypes are determined. capsular polysaccharide synthesis (CPS) genes are located at the cps locus. Production of the capsule is encoded by CPS genes (Smith et al., 1999). Especially, previous studies have shown that the *S. suis* serotype 2 mutant impaired in capsule expression acquire a biofilm-positive phenotype (Tanabe et al., 2010). Moreover, a hydrophilic capsule may hinder hydrophobic structures or components important for biofilm formation by *S. suis* (Bonifait et al., 2010).

Azithromycin is an antibiotic useful for the treatment of bacterial infections. It is common knowledge that azithromycin is derived from erythromycin, belonging to macrolide. Recently, some reports have showed that subinhibitory concentrations of azithromycin decreases biofilm formation (Starner et al., 2008; Gui et al., 2014). Our laboratory recently reported that sub-minimal inhibitory concentrations (MICs) of tylosin and sub-MICs of erythromycin decreased biofilm formation of *S. suis* ATCC700794 (Zhao et al., 2015; Wang et al., 2016). However, the relationship between azithromycin and biofilm formation by *S. suis* remains poorly understood. Therefore, the purpose of this study was to describe the use of proteomics to better understand the impact of azithromycin on *S. suis* biofilms *in vitro*. We found that azithromycin affected the CPS enzymes. Several cell surface proteins [such as ATP-binding cassette (ABC) superfamily ATP-binding cassette transporter (G7SD52), CpsR (K0FG35), Cps1/2H (G8DTL7), CPS16F (E9NQ13), putative uncharacterized protein (G7SER0), NADP-dependent glyceraldehyde-3-phosphate dehydrogenase (G5L259), putative uncharacterized protein (G7S2D6), amino acid permease (B0M0G6), and NsuB (G5L351)] were found to be implicated in biofilm formation. Especially, after growth with 1/2 MIC of azithromycin and 1/4 MIC of azithromycin, the capsular polysaccharide (CP) content of *S. suis* was significantly higher.

## Materials and Methods

### Bacterial Strains and Growth Conditions

*Streptococcus suis* ATCC700794 was used in this study. Bacteria were grown aerobically at 37°C in Todd-Hewitt broth (THB; Summus Ltd, Harbin, Heilongjiang, China) or Todd-Hewitt broth agar (THA) supplemented with 5% (v/v) fetal bovine serum (Sijiqing Ltd, Hangzhou, Zhejiang, China). The cultures were used for the biofilm assays and the MIC assays.

### Determination of Growth Inhibition Activity of Azithromycin

Minimal inhibitory concentration assays of azithromycin were done three times (refer to Yang et al., 2015), with a few modifications. Briefly, *S. suis* ATCC700794 was grown aerobically at 37°C in THB (Summus Ltd, Harbin, Heilongjiang, China) overnight. The overnight cultures were diluted in sterile physiological saline (corresponding to 1 × 10^8^ colony-forming units /ml). Then, dilute the cultures of *S. suis* ATCC700794 1:100 using sterile THB (Summus Ltd, Harbin, Heilongjiang, China). Finally, samples (100 μl) were added to the wells of a 96-well plate (Corning Costar^®^3599, Corning, NY, USA) containing serial dilutions of azithromycin in culture medium. Control bacteria were cultivated in the absence of azithromycin. The MIC was determined as the lowest concentration of azithromycin that completely inhibited *S. suis* growth after incubation for 24 h at 37°C.

The growth rates of *S. suis* ATCC700794 treated with 1/2 MIC of azithromycin and untreated *S. suis* ATCC700794 were analyzed (refer to Yang et al., 2015). Briefly, *S. suis* ATCC700794 treated without azithromycin and *S. suis* ATCC700794 treated with azithromycin (1/2 MIC) were incubated at 37°C for 12 h. Then, the samples were taken every hour for measuring OD 600 nm.

### Biofilm Assay

Overnight cultures of *S. suis* were diluted in sterile THB supplemented with 5% (v/v) fetal bovine serum (corresponding to 1 × 10^8^ colony-forming units/ml). Then, the diluted overnight cultures (200 μl) was grown (24 h) in a 96-well plate (Corning Costar^®^ 3599, Corning, NY, USA) in the presence of 1/2 MIC, 1/4 MIC, 1/8 MIC, or 1/16 MIC of azithromycin. *S. suis* ATCC700794 treated without azithromycin was served as a control. Biofilms were treated as described by Yang et al. (2015) with some modifications. Briefly, the medium, free-floating bacteria, and loosely bound biofilm were then removed by aspiration, and the wells were washed three times with sterile physiological saline. The remaining attached bacteria were fixed with 200 μl of 99% methanol (Guoyao Ltd, China) per well, and then the wells were left to dry. The wells were dyed for 30 min with 200 μl of 0.1% crystal violet (Sularbao Ltd, Beijing, China) per well. The stain was washed with water. Then, the dye was solubilized with 200 μl of 33% glacial acetic acid per well. Finally, the samples were measured the absorbance at 570 nm.

### Scanning Electron Microscopy

The biofilm structure of *S. suis* was observed by scanning electron microscopy (refer to Yang et al., 2015). Briefly, overnight cultures of *S. suis* were diluted in sterile THB supplemented with 5% (v/v) fetal bovine serum (corresponding to 1 × 10^8^ colony-forming units/ml). Then, the diluted overnight cultures with 1/2 MIC of azithromycin or without azithromycin were added into wells of a six-well plate containing rough glass slide or glass slide. Free-floating bacteria and medium were removed after 24 h in culture. The biofilms were incubated overnight in fixation buffer, washed with cacodylate buffer and post-fixed for 90 min at room temperature in 1% (w/v) osmic acid containing 2 mM potassium ferrocyanide and 6% (w/v) sucrose in cacodylate buffer. Samples were dehydrated through a graded series of ethanol (50, 70, 95, and 100%), critical point dried, gold sputtered and examined using a scanning electron microscopy.

### iTRAQ Analysis

Protein was extracted from *S. suis* cells at two different conditions (1/2 MIC of azithromycin-treated cells and non-treated cells). iTRAQ analysis was implemented at Shanghai Applied Protein Technology Co. Ltd (APT, Shanghai, China). Three biological replicates were evaluated to minimize the influence of less reliable quantitative information. iTRAQ analyses were performed as described by Zhao et al. (2015). Briefly, protein digestion was performed according to the FASP procedure and the resulting peptide mixture was labeled using the 8-plex iTRAQ reagent. iTRAQ-labeled peptides were fractionated by strong cation exchange (SCX) chromatography using the AKTA Purifier system. LC–MS/MS analysis was performed on a Q Exactive mass spectrometer that was coupled to Easy nLC (Proxeon Biosystems, now Thermo Fisher Scientific).

### Sequence Database Searching and Data Analysis

Sequence database searching and data analysis were implemented at Shanghai Applied Protein Technology Co. Ltd (APT, Shanghai, China). Briefly, MS/MS spectra were searched using MASCOT engine embedded into Proteome Discoverer 1.3 against Uniprot database (133549 sequences, download at March 3, 2013) and the decoy database. For protein identification, the following options were used. Peptide mass tolerance = 20 ppm, MS/MS tolerance = 0.1 Da, enzyme = Trypsin, missed cleavage = 2, fixed modification: Carbamidomethyl (C), iTRAQ8plex(K), iTRAQ8plex (N-term), and variable modification:oxidation (M), FDR ≤ 0.01.

### Quantitative RT-PCR Analysis

We investigated the effect of 1/2 MIC of azithromycin on the gene expression of CPS enzymes (CpsR, Cps1/2H, and CPS16F). *S. suis* was grown to mid-log phase and then the culture medium was supplemented with 1/2 MIC of azithromycin prior to further incubate at 37°C for 24 h. Control cells were incubated in the absence of azithromycin.

Quantitative RT-PCR were implemented as described by Yang et al. (2015).

Briefly, bacteria were collected by centrifugation (10,000 × *g* for 5 min) and treated with an RNASE REMOVER I (Huayueyang Ltd, Beijing, China). Total RNA was extracted with a Bacterial RNA isolating kit (Omega, Beijing, China) according to the manufacturer’s protocol. RNA was reverse transcribed using Maloney murine leukemia virus reverse transcriptase and random hexamers in a S1000^TM^ thermal cycler. Reverse transcription conditions were 90 min at 42°C and 15 min at 70°C. Real-time PCR was used for quantification of *cpsR, cps1/2H*, and *cps16F* mRNA expression. Relative copy numbers and expression ratios of selected genes were normalized to the expression of 16S rRNA gene (housekeeping gene). The 16S rRNA gene was used as an internal control for specific primers. The specific primers used for the quantitative RT-PCR were purchased from Takara and are listed in **Table 1**. Triplicate reactions were prepared with 25 μl of PCR mixture containing 12.5 μl of IQ SYBR Green Supermix, 5 μl of cDNA, 1 μl of gene-specific primer, and 6.5 μl of RNase- and DNase-free water. The samples were amplified using a Bio-Rad MyCycler^TM^ thermal cycler (Bio-Rad Laboratories). The amplification conditions for *cpsR, cps1/2H, cps16F*, and16S rRNA were 94°C for 10 min followed by 40 cycles at 94°C for 15 s, 60°C for 60 s.

**Table 1:** Primers used for the quantitative RT-PCR analysis.

Genes	Primer sequence
*cps1/2H*	Forward: 5′-GCCTCTTATTCAGGTTAT-3′ Reverse: 5′-GTTCTGCTACTGTTTCTC-3′
*cps16F*	Forward: 5′-TGAACATAATGGAGCAAC-3′ Reverse: 5′-AGGACCAATCCGACAAGC-3′
*cpsR*	Forward: 5′-TTCGGTAGTAGAAGGTTCAAGAC-3’ Reverse: 5′-AACACGCAAAGCTAAATAGGTAT-3′
*16s rRNA*	Forward: 5′-GATATATGGAGGAACACCG-3′ Reverse: 5′-GACCCAACACCTAGCACT-3′

### Quantitative Determination of CP

*Streptococcus suis* ATCC700794 was treated with 1/2 MIC, 1/4 MIC or 1/8 MIC of azithromycin. *S. suis* ATCC700794 treated without azithromycin was served as a control. Isolation and purification of the CP from *S. suis* ATCC700794 were performed as described by Yufang et al. (refer to Yufang et al., 2006). Briefly, a 10-ml overnight culture was used to inoculate 1L of THB containing 5% (v/v) fetal bovine serum for 24 h at 37°C. The bacterial cells were recovered by centrifugation and washed twice with PBS. The pellet was suspended in 100 ml of glycine buffer containing 100 mg lysozyme (Sigma). After incubation at 37^o^C for 8 h, the supernatant was recovered following centrifugation. The supernatant was treated with 20 mg of DNase (Sigma) and 10 mg of RNase (Sigma) at 37°C for 1 h. Proteinase K (20 mg) was added for 2 h at 55°C. The supernatant was brought to a concentration of 30% (v/v) ethanol and allowed to stand at 4°C for 2 h. The precipitate formed was removed by centrifugation, and the supernatant was brought to a concentration of 80% ethanol and allowed to stand at 4°C overnight. The precipitate was recovered by centrifugation and dried. Quantitative determination of the CP was performed as described by Cuesta et al. (2003). Briefly, the phenol solution (0.3 ml) and the CP solution (0.6 ml) were added to screw cap tubes, which were capped and vortex-stirred. Then 1.5 ml of concentrated sulfuric acid was added directly to the tube. The tubes were then closed, vortex-stirred for 5 s and incubated for 30 min. All samples were read the absorbances at 490 nm using distilled water as blank in a UV-7504 spectrophotometer (Jingke Ltd, Shanghai, China). Standard curve of glucose was performed as above described. The CP content was expressed according to the formula: CP content (%) = (*C* × *D* × *F/W*) × 100, where *C* is sugar concentration in the sample, *D* is dilution factor in the sample, *F* is conversion factor (3.19), and *W* is CP weight in the sample.

### Statistical Analysis

Assays were done three times and the means ± standard deviations were computed. Data were analyzed using the Student *t*-test.

## Results

**FIGURE 1:**
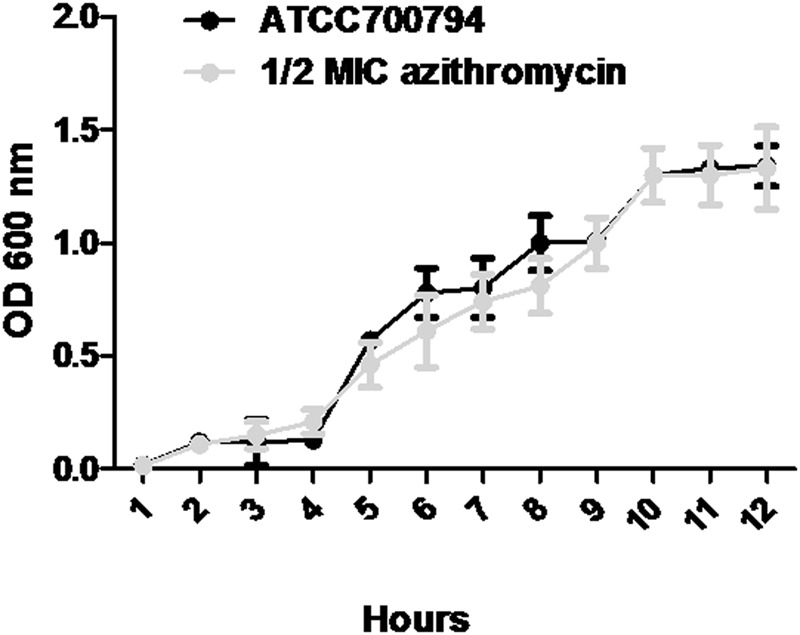
**Growth curve of ATCC700794 in the absence of azithromycin and in the presence of 1/2 minimal inhibitory concentration (MIC) of azithromycin.** Data are expressed as means ± standard deviations.

**FIGURE 2:**
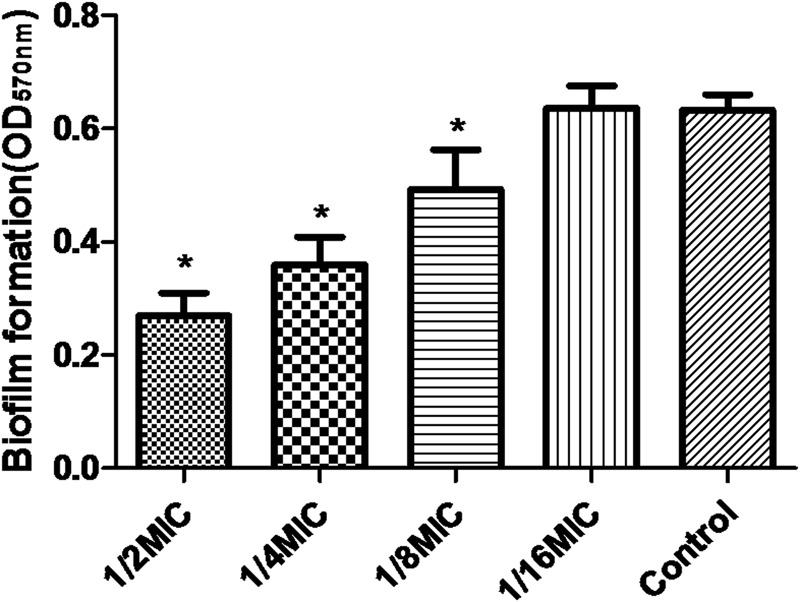
**Effect of sub-MICs of azithromycin on biofilm formation by *Streptococcus suis* ATCC700794.** Data are expressed as means ± standard deviations. Controls refer to the absence of azithromycin. Significantly different (^∗^*p* < 0.05) compared to untreated control bacteria.

Minimal inhibitory concentration of azithromycin against *S. suis* ATCC700794 was 32 μg/ml in the present study. Furthermore, after 10 h incubation at 37°C, untreated *S. suis* ATCC 700794 and treated (1/2 MIC of azithromycin) *S. suis* ATCC 700794 reached stationary phase, indicating no effect on growth rate of *S. suis* ATCC700794 (**Figure 1**). After growth without azithromycin (control) or with 1/2 MIC of azithromycin, 1/4 MIC of azithromycin, 1/8 MIC of azithromycin, or 1/16 MIC of azithromycin, biofilm formation of *S. suis* was investigated (**Figure 2**). When the culture medium was supplemented with 1/2 MIC of azithromycin, 1/4 MIC of azithromycin, or 1/8 MIC of azithromycin, the biofilms of *S. suis* were significantly lower in comparison with the control (*p* < 0.05). However, after growth with 1/16 MIC of azithromycin, the biofilms were not significantly affected (*p* > 0.05).

**FIGURE 3:**
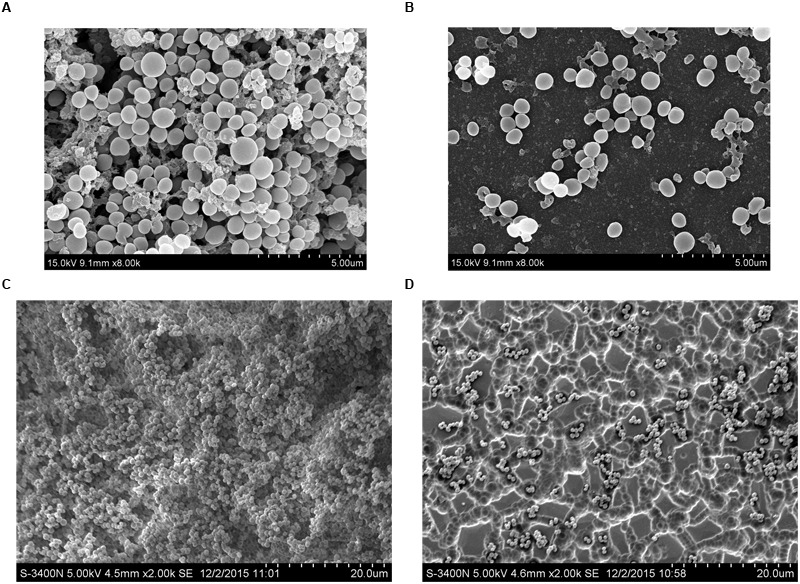
**Scanning electron micrographs of *S. suis* ATCC700794 biofilm following growth in THB supplemented without azithromycin [**(A or C)**, control], or with 1/2 MIC of azithromycin **(B or D)**.** Controls refer to the absence of azithromycin.

Scanning electron microscopy analysis was performed to observe the azithromycin sub-MIC-induced biofilm formation by *S. suis*. As shown in **Figures 3A,C** (control), a thick biofilm made of aggregates and microcolonies almost completely covered the surface of the glass slide. However, when the culture medium was supplemented with 1/2 MIC of azithromycin, individual short chains of *S. suis* and individual pairs of *S. suis* attached to the glass slide or rough glass slide (**Figures 3B,D**). Scanning electron microscopy analysis revealed that 1/2 MIC of azithromycin significantly decreased biofilm formation by *S. suis*.

iTRAQ technology was used to compare the patterns of protein expression at two different conditions (1/2 MIC of azithromycin treated cells and non-treated cells). When a protein had both a fold-change of more that a ratio >2.0 or <0.5 (*p*-value < 0.05), the protein was considered differentially expressed. On the basis of the two criteria, 19 differentially expressed proteins were identified, 12 (63.2%) of which displayed increased, and 7 (36.8%) displayed decreased abundance (**Table 2**). According to the molecular function, these proteins were classified into following categories: nucleotide binding (4, 21.1%), DNA binding (1, 5.3%), catalytic activity (7, 36.8%), RNA binding (1, 5.3%), protein binding (1, 5.3%), structural molecule activity (1, 5.3%), transporter activity (1, 5.3%), motor activity (1, 5.3%), and unknown molecular function (9, 47.4%). According to the cellular component, these proteins were classified into following categories: membrane (9, 47.4%), cytoplasm (2, 10.5%), extracellular (1, 5.3%), cytoskeleton(1, 5.3%), and unknown cellular component (9, 47.4%). According to the biological process, these proteins were classified into following categories: metabolic process (5, 26.3%), cell organization and biogenesis (1, 5.3%), regulation of biological process (1, 5.3%), transport (2, 10.5%), cellular homeostasis (1, 5.3%), and unknown biological process (12, 63.2%). Detailed information can be found in Supplementary Table S1. The majority of the proteins were involved in catalytic activity (7, 36.8%) and metabolism (5, 26.3%). These findings suggested that 1/2 MIC of azithromycin-treated cells and non-treated cells were subjected to different pharmic selective pressures, which might result in different proteome patterns.

**Table 2:** ITRAQ Identification of differentially expressed proteins.

Accession	Proteins	Fold change^a^
**Up-regulated proteins**
G5L351	NsuB	7.12934739263001
G5KZF5	Putative uncharacterized protein	3.01653085375799
G8DTL7	Cps1/2H	2.02407561418484
G7SFA8	Transcriptional regulator, Cro/CI family	2.57596261219689
G7S2D6	Putative uncharacterized protein	3.00076898311981
Q9EZW2	Elongation factor Tu (Fragment)	3.9674279008791
G7S4L6	Putative uncharacterized protein	2.31735729535592
B0M0G6	Amino acid permease (Fragment)	2.16331972961307
A4W361	Uridine kinase	7.48970780907174
R4NZ92	TPR repeat-containing protein	2.70348016201752
G7SD52	ABC superfamily ATP binding cassette transporter, membrane protein	5.6411425449412
G7S7A9	FAD-dependent pyridine nucleotide-disulfide oxidoreductase	2.96465802924635
**Down-regulated proteins**
G7SER0	Putative uncharacterized protein	0.206326492539119
G7SHZ3	Bacteriophage protein, putative	0.480729105006118
K0FG35	CpsR	0.369579660174813
G5L259	NADP-dependent glyceraldehyde-3-phosphate dehydrogenase, putative	0.374165986672724
G7RZ18	Sortase-like protein	0.368308683014746
E9NQ13	CPS16F	0.24025009068188
G7SPA1	Putative scaffolding protein	0.324624727229277

*^a^1/2 MIC azithromycin treated vs. non-treated cells.*

**FIGURE 4:**
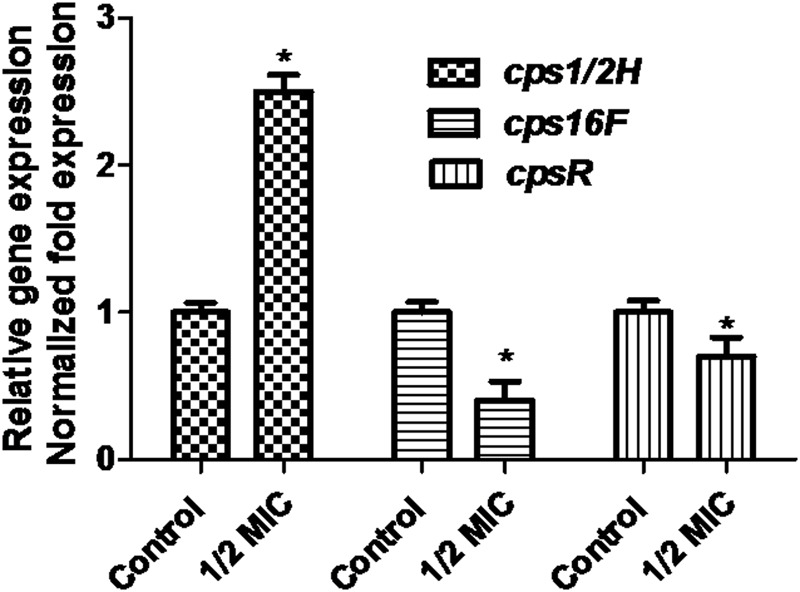
**Relative abundances of *cps1/2H, cps16F* and *cpsR* in 1/2 MIC azithromycin-treated and -untreated cell revealed by quantitative RT-PCR.** Data are expressed as means ± standard deviations. The expression was normalized to 16S rRNA. Controls refer to the absence of azithromycin. Significantly different (^∗^*p* < 0.05) compared to untreated control bacteria.

**FIGURE 5:**
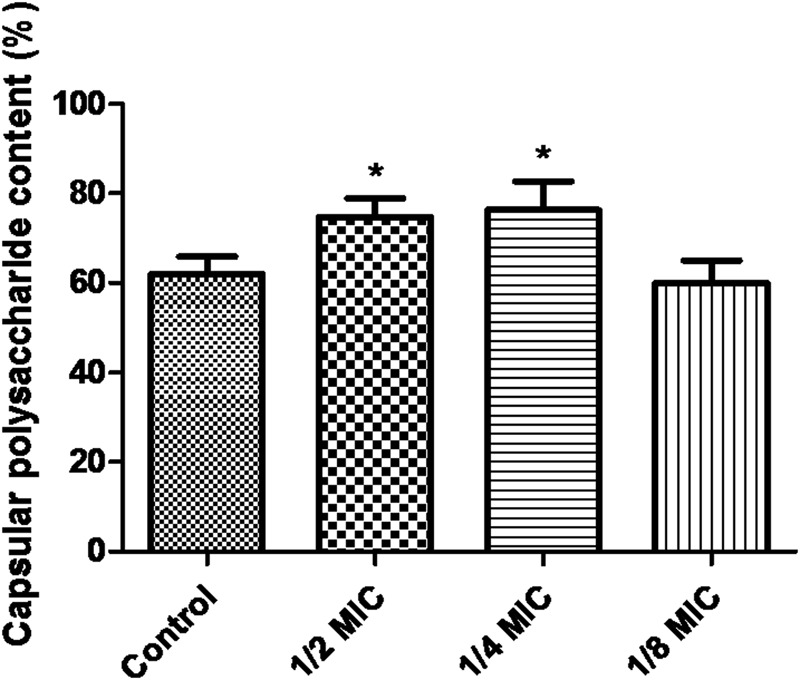
**Determination of capsular polysaccharide content of *S. suis* ATCC700794 using a phenol–sulfuric acid method.** Data are expressed as means ± standard deviations. Controls refer to the absence of azithromycin. Significantly different (^∗^*p* < 0.05) compared to untreated control bacteria.

When the culture medium was supplemented with 1/2 MIC of azithromycin, expression of the genes *cps1/2H* was upregulated (*p* < 0.05; **Figure 4**). However, as shown in **Figure 4**, when the culture medium was supplemented with 1/2 MIC of azithromycin, expression of the genes *cpsR* and *cps16F* was downregulated (*p* < 0.05). As shown in **Figure 5**, the CP content, in comparison with the control, was significantly higher (*p* < 0.05) after growth with 1/2 MIC and 1/4 MIC of azithromycin. However, the CP content was not significantly affected following growth in the presence of 1/8 MIC of azithromycin (*p* > 0.05).

## Discussion

In the present study, the relationship between biofilm formation of *S. suis* and azithromycin have been investigated carefully. Some researchers have reported that subinhibitory concentrations of antimicrobial agents (such as moxifloxacin and triclosan) can either decrease (Pompilio et al., 2010) or increase (Bedran et al., 2014) biofilm formation of bacterial pathogens. In the present study, we found that subinhibitory concentrations of azithromycin significantly decreased biofilm formation of *S. suis*. This finding is consistent with the previous publication (Starner et al., 2008; Gui et al., 2014).

Cell surface proteins and outer membrane proteins play a crucial role in biofilm formation of bacterial pathogens. Biofilm formation of bacterial pathogens mainly included three aspects: (i) bacterial pathogens adhere to surfaces, (ii) aggregation of micro-colonies of bacterial pathogens, and (iii) further expansion of the microbial community. Key stage of biofilm formation of bacterial pathogens is adhesion to surfaces. Cell surface proteins and outer membrane proteins may mediate cell attachment to surfaces.

When bacterial cells and a surface are close to each other, the biophysical interaction between a surface and bacterial cells plays an important role in adhesion. Therefore, cell surface proteins and outer membrane proteins play an important role in biofilm formation of bacterial pathogens. Membrane proteins such as OmpA mediate cell adhesion in *Acinetobacter baumannii* (Dallo et al., 2010). In the present study, the expression of ABC superfamily ATP binding cassette transporter (G7SD52), CpsR (K0FG35), Cps1/2H (G8DTL7), CPS16F (E9NQ13), putative uncharacterized protein (G7SER0), NADP-dependent glyceraldehyde-3-phosphate dehydrogenase (G5L259), putative uncharacterized protein (G7S2D6), amino acid permease (B0M0G6), and NsuB (G5L351) changed; these proteins belong to membrane proteins and cell-surface proteins and might be involved in some molecular functions including transporter activity, nucleotide binding, motor activity, protein binding and catalytic activity (Supplementary Table S1). We predicted that these membrane proteins might affect the bacterial cell-cell interaction. Thus, membrane proteins might play a significant role in biofilm formation.

Interestingly, ABC superfamily ATP-binding cassette transporter (G7SD52) and NsuB (G5L351) was up-regulated in sub-MIC of azithromycin (in this study) and erythromycin-treated *S. suis* (Zhao et al., 2015). However, molecular function and biological process of NsuB (G5L351) are currently unknown. In future, NsuB (G5L351) should be studied. CpsR (K0FG35) and CPS16F (E9NQ13) was down-regulated in sub-MIC of azithromycin (in this study) and erythromycin-treated *S. suis* (Zhao et al., 2015). Especially, in the present study, azithromycin also affected the expression of the other proteins, for example, Putative uncharacterized protein (G7SER0), NADP-dependent glyceraldehyde-3-phosphate dehydrogenase (G5L259), putative uncharacterized protein (G7S2D6), and amino acid permease (B0M0G6). This finding is not consistent with the previous results of our laboratory showing that subinhibitory concentrations of erythromycin inhibit biofilm formation of *S. suis* (Zhao et al., 2015). It is generally known that azithromycin is the same class antibiotics as erythromycin, belonging to macrolide. However, erythromycin did not affect the expression of the four proteins (Zhao et al., 2015). In future, this finding should be studied carefully.

ABC transporters are common proteins in bacteria. They often transport a variety of substrates (for example polypeptides, ions and nutrients) (Lin et al., 2014). ABC transporters are also presumably involved in the export of the competence-stimulating peptide (autoinducers), which in turn regulate expression of specific target genes (Li et al., 2002). Moreover, previous studies have also demonstrated that ABC transporters are important as they regulate biofilm formation (Selvaraj et al., 2014). Shannon et al. have reported that ABC transporters are required for biofilm formation by *Pseudomonas fluorescens* WCS365 (Hinsa et al., 2003). Our laboratory recently reported that ABC superfamily ATP-binding cassette transporter (G7SD52) was up-regulated in sub-MIC of erythromycin or sub-MIC of tylosin-treated *S. suis* (Zhao et al., 2015; Wang et al., 2016). In the present study, ABC superfamily ATP-binding cassette transporter (G7SD52) was upregulated in sub-MIC of azithromycin-treated cell (treated vs. untreated). However, azithromycin did not affect the expression of histidine kinase of the competence regulon (comD; G5L3D2) and response regulator (comE; G7S4A2) in the present study. Interestingly, erythromycin affects the expression of the two proteins (Zhao et al., 2015). Li et al. (2002) have also found that comD and comE are implicated in biofilm formation of *S. mutans*. So far, the detailed molecular mechanism was still unknown.

Some researchers have reported that the *S. suis* mutant impaired in capsule expression acquire a biofilm-positive phenotype in the previous publication (Tanabe et al., 2010). Bonifait et al. have reported that a hydrophilic capsule hinder hydrophobic components or structures. Moreover, Bonifait et al. (2010) have also found that the hydrophobic components or structures play an important role in biofilm formation of *S. suis*. Non-encapsulated *S. suis* strains may form thick biofilms (Benga et al., 2004). Specifically, in other species, Qin et al. (2013) have reported that reduced *S. pneumoniae* CP may increase biofilm development. Joseph et al. reported that *Vibrio vulnificus* CP expression actually inhibited biofilm formation (Joseph and Wright, 2004). Lakkitjaroen et al. (2014) have reported that the inactivation of Cps2F (glycosyltransferases) often caused capsule loss in *S. suis*. Cps2F and Cps2H may be involved in the biosynthesis of serotype 2 CP. In this study, azithromycin affected the biosynthesis of the CPS enzymes, for example, CpsR (K0FG35), Cps1/2H (G8DTL7), and CPS16F (E9NQ13). This finding is consistent with the previous results of our laboratory showing that sub-MIC erythromycin inhibits biofilm formation of *S. suis* (Zhao et al., 2015). However, Cps1/2H (G8DTL7) was downregulated (fold change: 0.30) in sub-MIC of erythromycin-treated *S. suis* (Zhao et al., 2015). Interestingly, Cps1/2H (G8DTL7) was up-regulated (fold change: 2.024) in sub-MIC of azithromycin-treated *S. suis* (in this study). It is common knowledge that azithromycin is derived from erythromycin, belonging to macrolide. We speculated that azithromycin had different molecule structure in comparison with erythromycin, which in turn might generate the different proteome patterns. However, the detailed molecular mechanism was still unknown. In future, this finding should be studied carefully. In brief, we speculated that azithromycin might affect the biosynthesis of the CP.

Recently, Lee et al. have investigated the role of CP in biofilm formation in *V. vulnificus*. *V. vulnificus* mutant impaired in CP expression increases cell aggregation, adherence to abiotic surfaces, and hydrophobicity of the cell surface. In comparison with wild type, *V. vulnificus* mutant forms significantly more biofilm. Furthermore, addition of CP decrease biofilms formation of a CP-deficient mutant. After the CP-producing wild type is degraded endogenous CP with an enzyme, the CP-producing wild type increases biofilm formation (Lee et al., 2013). In the present study, the CP content, in comparison with the control, was significantly higher after growth with 1/2 MIC of azithromycin and 1/4 MIC of azithromycin. Houde et al. have reported that *S. suis* mutants impaired in capsule expression are easily killed by immune cells. Therefore, CP of *S. suis* is a significant virulence factor of the bacteria. Furthermore, CP is also involved in adhesion of the bacteria (Houde et al., 2012). Key stage of biofilm formation of bacterial pathogens is adhesion to surfaces. We speculated that azithromycin might affect adhesion of *S. suis* by increasing capsular polysaccharide content. In brief, further examination of the relationship of capsule structure to biofilms should be studied.

In short, subinhibitory concentrations of azithromycin might decrease biofilm formation of *S. suis* in the present study. At two different conditions (1/2 MIC of azithromycin non-treated cells and treated cells), we carried out comparative proteomic analyses of cells by using iTRAQ technology. In the present study, 19 differentially expressed proteins were identified, 12 (63.2%) of which displayed increased and 7 (36.8%) displayed decreased abundance. Several cell surface proteins [such as ABC superfamily ATP-binding cassette transporter (G7SD52), CpsR (K0FG35), Cps1/2H (G8DTL7), CPS16F (E9NQ13), putative uncharacterized protein (G7SER0), NADP-dependent glyceraldehyde-3-phosphate dehydrogenase (G5L259), putative uncharacterized protein (G7S2D6), Amino acid permease (B0M0G6), and NsuB (G5L351)] were found to be implicated in biofilm formation. It is common knowledge that azithromycin is derived from erythromycin, belonging to macrolide. We speculated that azithromycin had different molecule structure in comparison with erythromycin, which in turn might generate the different proteome patterns. We also discovered that azithromycin might affect the biosynthesis of the CP. These data showed a useful starting point for more focused studies to understand what exactly was going on. However, the detailed mechanism was still unknown. In future, the relationship between capsule structure and biofilms of *S. suis* should be studied to delineate the complex mechanism.

## Author Contributions

Y-BY designed the whole experiment. Y-HL directed the completion of the experiment. J-QC, Y-LZ, J-WB, W-YD, Y-HZ, X-YC, and DL provided help during the experiment.

## Conflict of Interest Statement

The authors declare that the research was conducted in the absence of any commercial or financial relationships that could be construed as a potential conflict of interest.

## References

[B1] Bedran T. B. L. Grignon L. Spolidorio D. P. Grenier D. (2014). Subinhibitory concentrations of triclosan promote *Streptococcus mutans* biofilm formation and adherence to oral epithelial cells. *PLoS ONE* 9:e89059 10.1371/journal.pone.0089059 PMC3923858 24551218

[B2] Benga L. Goethe R. Rohde M. Valentin-Weigand P. (2004). Non-encapsulated strains reveal novel insights in invasion and survival of *Streptococcus suis* in epithelial cells. *Cell. Microbiol.* 6 867–881. 10.1111/j.1462-5822.2004.00409.x 15272867

[B3] Bojarska A. Molska E. Janas K. Skoczynska A. Stefaniuk E. Hryniewicz W. (2016). *Streptococcus suis* in invasive human infections in Poland: clonality and determinants of virulence and antimicrobial resistance. *Eur. J. Clin. Microbiol. Infect. Dis.* 35 917–925. 10.1007/s10096-016-2616-x 26980093 PMC4884564

[B4] Bonifait L. Gottschalk M. Grenier D. (2010). Cell surface characteristics of nontypeable isolates of *Streptococcus suis*. *FEMS Microbiol. Lett.* 311 160–166. 10.1111/j.1574-6968.2010.02086.x 20738400

[B5] Cuesta G. Suarez N. Bessio M. I. Ferreira F. Massaldi H. (2003). Quantitative determination of pneumococcal capsular polysaccharide serotype 14 using a modification of phenol-sulfuric acid method. *J. Microbiol. Methods* 52 69–73. 10.1016/s0167-7012(02)00151-3 12401228

[B6] Dallo S. F. Denno J. Hong S. Weitao T. (2010). Adhesion of *Acinetobacter baumannii* to extracellular proteins detected by a live cell-protein binding assay. *Ethn. Dis.* 20 7–11. 20521377

[B7] Espinosa I. Baez M. Lobo E. Martinez S. Gottschalk M. (2016). Antimicrobial activity of penicillin G and N-acetylcystein on planktonic and sessile cells of *Streptococcus suis*. *Pol. J. Microbiol.* 65 105–109. 10.5604/17331331.1197282 27282001

[B8] Gui Z. H. Wang H. F. Ding T. Zhu W. Zhuang X. Y. Chu W. H. (2014). Azithromycin reduces the production of alpha-hemolysin and biofilm formation in *Staphylococcus aureus*. *Indian J. Microbiol.* 54 114–117. 10.1007/s12088-013-0438-4 24426177 PMC3889843

[B9] Hinsa S. M. Espinosa-Urgel M. Ramos J. L. O’Toole G. A. (2003). Transition from reversible to irreversible attachment during biofilm formation by *Pseudomonas fluorescens* WCS365 requires an ABC transporter and a large secreted protein. *Mol. Microbiol.* 49 905–918. 10.1046/j.1365-2958.2003.03615.x 12890017

[B10] Houde M. Gottschalk M. Gagnon F. Van Calsteren M. R. Segura M. (2012). *Streptococcus suis* capsular polysaccharide inhibits phagocytosis through destabilization of lipid microdomains and prevents lactosylceramide-dependent recognition. *Infect. Immun.* 80 506–517. 10.1128/iai.05734-11 22124659 PMC3264306

[B11] Joseph L. A. Wright A. C. (2004). Expression of *Vibrio vulnificus* capsular polysaccharide inhibits biofilm formation. *J. Bacteriol.* 186 889–893. 10.1128/jb.186.3.889-893.2004 14729720 PMC321485

[B12] Lakkitjaroen N. Takamatsu D. Okura M. Sato M. Osaki M. Sekizaki T. (2014). Capsule loss or death: the position of mutations among capsule genes sways the destiny of *Streptococcus suis*. *FEMS Microbiol. Lett.* 354 46–54. 10.1111/1574-6968.12428 24654559

[B13] Lee K. J. Kim J. A. Hwang W. Park S. J. Lee K. H. (2013). Role of capsular polysaccharide (CPS) in biofilm formation and regulation of CPS production by quorum-sensing in *Vibrio vulnificus*. *Mol. Microbiol.* 90 841–857. 10.1111/mmi.12401 24102883

[B14] Li Y.-H. Tang N. Aspiras M. B. Lau P. C. Y. Lee J. H. Ellen R. P. (2002). A quorum-sensing signaling system essential for genetic competence in *Streptococcus mutans* is involved in biofilm formation. *J. Bacteriol.* 184 2699–2708. 10.1128/jb.184.10.2699-2708.2002 11976299 PMC135014

[B15] Lin C. H. Su S. C. Ho K. H. Hsu Y. W. Lee K. R. (2014). Bactericidal effect of sulbactam against *Acinetobacter baumannii* ATCC 19606 studied by 2D-DIGE and mass spectrometry. *Int. J. Antimicrob. Agents* 44 38–46. 10.1016/j.ijantimicag.2014.03.004 24837410

[B16] Nakamura Y. Yamamoto N. Kino Y. Yamamoto N. Kamei S. Mori H. (2016). Establishment of a multi-species biofilm model and metatranscriptomic analysis of biofilm and planktonic cell communities. *Appl. Microbiol. Biotechnol.* 100 7263–7279. 10.1007/s00253-016-7532-6 27102130

[B17] Pompilio A. Catavitello C. Picciani C. Confalone P. Piccolomini R. Savini V. (2010). Subinhibitory concentrations of moxifloxacin decrease adhesion and biofilm formation of *Stenotrophomonas maltophilia* from cystic fibrosis. *J. Med. Microbiol.* 59 76–81. 10.1099/Jmm.0.011981-0 19762476

[B18] Qin L. Kida Y. Imamura Y. Kuwano K. Watanabe H. (2013). Impaired capsular polysaccharide is relevant to enhanced biofilm formation and lower virulence in *Streptococcus pneumoniae*. *J. Infect. Chemother.* 19 261–271. 10.1007/s10156-012-0495-3 23229613

[B19] Selvaraj A. Sumantran V. Chowdhary N. Kumar G. R. (2014). Prediction and classification of ABC transporters in *Geobacter sulfurreducens* PCA using computational approaches. *Curr. Bioinform.* 9 166–172. 10.2174/1574893608999140109113236

[B20] Smith H. E. Damman M. van der Velde J. Wagenaar F. Wisselink H. J. Stockhofe-Zurwieden N. (1999). Identification and characterization of the cps locus of *Streptococcus suis* serotype 2: the capsule protects against phagocytosis and is an important virulence factor. *Infect. Immun.* 67 1750–1756. 10085014 10.1128/iai.67.4.1750-1756.1999 PMC96524

[B21] Starner T. D. Shrout J. D. Parsek M. R. Appelbaum P. C. Kim G. (2008). Subinhibitory concentrations of azithromycin decrease nontypeable *Haemophilus influenzae* biofilm formation and diminish established biofilms. *Antimicrob. Agents Chemother.* 52 137–145. 10.1128/aac.00607-07 17954687 PMC2223912

[B22] Tanabe S. I. Bonifait L. Fittipaldi N. Grignon L. Gottschalk M. Grenier D. (2010). Pleiotropic effects of polysaccharide capsule loss on selected biological properties of *Streptococcus suis*. *Can. J. Vet. Res.* 74 65–70. 20357962 PMC2801315

[B23] Wang S. Yang Y. B. Zhao Y. L. Zhao H. H. Bai J. W. Chen J. Q. (2016). Sub-MIC tylosin inhibits *Streptococcus suis* biofilm formation and results in differential protein expression. *Front. Microbiol.* 7:384 10.3389/fmicb.2016.00384 PMC4811924 27065957

[B24] Wang Y. Yi L. Wang S. H. Fan H. J. Ding C. Mao X. (2015). Crystal structure and identification of two key amino acids involved in AI-2 production and biofilm formation in *Streptococcus suis* LuxS. *PLoS ONE* 10:e0138826 10.1371/journal.pone.0138826 PMC4618692 26484864

[B25] Yang Y. B. Wang S. Wang C. Huang Q. Y. Bai J. W. Chen J. Q. (2015). Emodin affects biofilm formation and expression of virulence factors in *Streptococcus suis* ATCC700794. *Arch. Microbiol.* 197 1173–1180. 10.1007/s00203-015-1158-4 26446827

[B26] Yufang Y. Linlin L. Xuenan S. U. N. Shuxue S. U. N. Zhaolin D. (2006). The isolation and purification of the capsular polysaccharide from group B streptococcus. *Chin. J. Biochem. Pharm.* 27 166–169.

[B27] Zhao Y. L. Zhou Y. H. Chen J. Q. Huang Q. Y. Han Q. Liu B. (2015). Quantitative proteomic analysis of sub-MIC erythromycin inhibiting biofilm formation of S-suis in vitro. *J. Proteom.* 116 1–14. 10.1016/j.jprot.2014.12.019 25579403

